# Genetic Information Insecurity as State of the Art

**DOI:** 10.3389/fbioe.2020.591980

**Published:** 2020-12-08

**Authors:** Garrett J. Schumacher, Sterling Sawaya, Demetrius King, Aaron J. Hansen

**Affiliations:** ^1^GeneInfoSec Inc., Boulder, CO, United States; ^2^Technology, Cybersecurity and Policy Program, College of Engineering and Applied Science, University of Colorado Boulder, Boulder, CO, United States; ^3^Department of Computer Science, College of Engineering and Applied Science, University of Colorado Boulder, Boulder, CO, United States

**Keywords:** biotechnology, cyberbiosecurity, cybersecurity, genomics, laboratory, cloud services, databases, privacy

## Abstract

Genetic information is being generated at an increasingly rapid pace, offering advances in science and medicine that are paralleled only by the threats and risk present within the responsible systems. Human genetic information is identifiable and contains sensitive information, but genetic information security is only recently gaining attention. Genetic data is generated in an evolving and distributed cyber-physical system, with multiple subsystems that handle information and multiple partners that rely and influence the whole ecosystem. This paper characterizes a general genetic information system from the point of biological material collection through long-term data sharing, storage and application in the security context. While all biotechnology stakeholders and ecosystems are valuable assets to the bioeconomy, genetic information systems are particularly vulnerable with great potential for harm and misuse. The security of post-analysis phases of data dissemination and storage have been focused on by others, but the security of wet and dry laboratories is also challenging due to distributed devices and systems that are not designed nor implemented with security in mind. Consequently, industry standards and best operational practices threaten the security of genetic information systems. Extensive development of laboratory security will be required to realize the potential of this emerging field while protecting the bioeconomy and all of its stakeholders.

## Introduction

Genetic information contained in nucleic acids, such as deoxyribonucleic acid (DNA), has become ubiquitous in society, enabled primarily by rapid biotechnological development and drastic decreases in DNA sequencing and DNA synthesis costs (Naveed et al., 2015; Berger and Schneck, 2019). Innovation in these industries has far outpaced regulatory capacity and remained somewhat isolated from the information security and privacy domains. Human genetic data contains a wealth of sensitive information. It can be used to identify an individual (Lin et al., 2004; Lowrance and Collins, 2007; Erlich et al., 2018) and predict their physical characteristics (Lippert et al., 2017; Li et al., 2019). The identifiability of genetic information is a critical challenge leading to growing consumer privacy concerns (Baig et al., 2020). Yet, genetic data is not always defined as protected health information or personally identifiable data by law. Once digital genetic data is stolen or disclosed, it cannot be reissued or changed in the same manner as other information types. A single human whole genome sequence can cost hundreds to thousands of dollars per sample, and when amassed, genetic information of large cohorts can be worth millions of dollars^1^ ^,2^ ^,3^. This positions human genetic information systems as likely targets for cyber and physical attacks, both of which could lead to global-scale impact.

It is also well known that biotechnology has a dual use nature leading to positive and negative applications, and genetic data of non-human sources is also valuable and can be considered sensitive. Synthetic biology has great potential to revolutionize many industries, but designer microbes can also be generated with CRISPR-Cas and other techniques that present global health and national security concerns (Salerno and Koelm, 2002; Chosewood and Wilson, 2009; Berger and Roderick, 2014; Werner, 2019). Microbiological genetic information systems are considered critical public health infrastructure (Fayans et al., 2020), plants can be manipulated to create potential health hazards (Mueller, 2019a), and methods for tracking genetically modified organisms can be exploited if appropriate techniques are not used (Mueller, 2019b). Sensitive genetic data of humans and other entities and their respective systems must be secured to prevent private to global risks (Jordan et al., 2020; Sawaya et al., 2020).

Security incidents surrounding genetic information systems are on the rise, and many relevant incidents have been documented by news sources^4^ ^,5^ ^,6^ ^,7^ and breach notifications^8^ ^,9^ ^,10^ ^,11^ ^,12^ ^,13^ ^,14^ ^,15^. The most common reasons have been misconfigurations in cloud security settings, email phishing attacks, and the compromise of connected third-party systems. As a result, these groups may face legal action^16^, penalties, reputational loss, and many other risks and consequences. The National Health Service’s Genomics England database in the United Kingdom has been targeted by nation-state threat actors^17^, and 23andMe’s Chief Security Officer said their database of around 10 million individuals is of extreme value and therefore “certainly is of interest to nation states.”^18^ Despite this recognition, proper measures to protect genetic information are often lacking under current practices in relevant industries and stakeholders.

Extensive work has been published surrounding the security of genetic information, highlighting that, as a newly developing field, cyberbiosecurity will require continuous assessment of risks as they emerge (Peccoud et al., 2018). Genetic information security is considered a critical aspect to comprehensive cyberbiosecurity and the bioeconomy (Institute of Medicine and National Research Council, 2006; Murch et al., 2018; Berger and Schneck, 2019; Murch and DiEuliis, 2019; Reed and Dunaway, 2019; Fayans et al., 2020; Jordan et al., 2020; National Academies of Sciences, Engineering, and Medicine, 2020; Sawaya et al., 2020). Multi-stakeholder and interdisciplinary collaboration, improved understanding of the security risks to biotechnology, characterization of biotechnology ecosystems, and assessment frameworks specific to biotechnology sectors and facility types will all be required in order to develop appropriate cyberbiosecurity countermeasures (Peccoud et al., 2018; Millett et al., 2019; Schabacker et al., 2019).

Toward the above issues and goals, this paper expands upon a previous microbiological genetic information system assessment (Fayans et al., 2020) by including a broader range of genetic information and system components, as well as novel concepts and additional vulnerabilities and threats to the ecosystem. Herein, genetic information systems are characterized from a security perspective, and the foundation for future assessments of these ecosystems has been established for which improvement and further development will be needed.

## Methodology

Confidential communications and interviews with leaders and technical personnel from eighteen relevant stakeholders occurred over the course of 9 months. These organizations can be broadly categorized as manufacturers and vendors, insurance and healthcare providers, research institutions, government and military groups, third-party service providers, and diagnostic laboratories. A third of these organizations contained one or more sequencing laboratories, and the remainder covered critical components of the system before or after sequencing laboratory stages. Several of the organizations allowed on-premise observation of, and interaction within, their environments, as well as in-depth uncredentialed and credentialed assessments of their property, people, processes, and technology. Specifically, DNA sequencing instruments as the point of raw data generation and other laboratory equipment and their networked data communications were focused on. Standard security tools and techniques were applied, such as vulnerability scanning, packet monitoring, threat modeling, configuration assessment, digital forensics, and full-stack assessments, including hardware teardowns and dynamic and static analysis of various software components. Organizational policies, external regulations, and other relevant items were also examined. Specific details and results have been omitted for confidentiality purposes. Such activities provided insight into the stakeholders’ perceptions, external requirements, implementations, concerns, and weaknesses regarding the security of their genetic information systems and organizations overall. This manuscript is primarily a summary of the researchers’ practical experience and direct observation of laboratory infrastructure backed by literature and industry input. Observed vulnerabilities and threats uncovered in the research have been reported to the appropriate agencies and stakeholders; this information will be made public once ethical disclosure and mitigation processes have concluded.

## The Genetic Information Threat Landscape

Confidentiality, integrity, and availability are the core principles governing the security of sensitive systems and information (International Organization for Standardization [ISO], 2012). Confidentiality is the principle of ensuring access to assets is restricted based upon the assets’ sensitivity. Integrity is the concept of protecting assets from unauthorized modification or deletion, while availability ensures assets are accessible to authorized parties at all times. Genetic information, which includes both biological material and digital genetic data, is the primary asset of concern, and associated assets, such as metadata, electronic health records and intellectual property, are also vulnerable within these systems. Genetic information systems are centered around one or more genetic sequencing devices, and include all inputs and outputs of these sequencing devices, as well as all upstream or downstream components that handle those data or materials.

**FIGURE 1:**
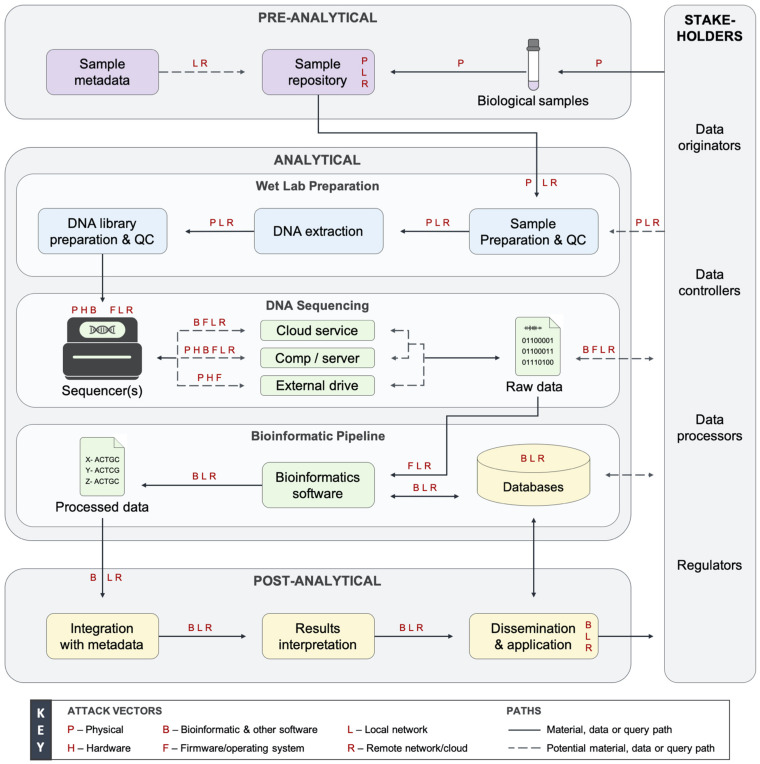
Data flow diagram of a generalized genetic information system and the accompanying threat landscape. Genetic information systems are cyber-physical systems divided into three phases with people interacting with system components throughout. The pre-analytical phase involves the collection, storage, and distribution of biological samples. The analytical phase includes wet laboratory preparation, DNA sequencing, and bioinformatic pipeline subphases. In the analytical phase, genetic data is generated, analyzed, transmitted, and stored by several hosts or devices. The post-analytical phase involves the dissemination, application, and amassed long-term storage of genetic data. Every system component and stakeholder are vulnerable to exploitation via the attack vectors denoted by red letters. Figure modified from Fayans et al. (2020) with permissions. More information on the ecosystem is provided in Supplementary Appendix 2. QC, quality control; Comp, computing device.

Genetic information systems are distributed cyber-physical systems containing numerous stakeholders (Supplementary Appendix 1), personnel, and devices with extensive computing and networking capabilities (Reed and Dunaway, 2019; Figure 1). Software, hardware, and many other components introduce attack vectors that can be used to compromise these systems (Figure 1), including through purposefully adversarial activity and human error. Organizations take steps to monitor and prevent error, and molecular biologists are skilled in laboratory techniques; however, they were found to commonly not have the expertise and resources to securely configure and operate these environments, nor are stakeholders always enabled to do so by third-party service contracts that we examined. Basic security features and tools, such as antivirus software, are usually recommended with little support given, and they can also easily be subverted. Advanced and comprehensive controls and policies are not commonly implemented. On-premise or adjacent network attacks could lead to certain devices, stakeholders, and individuals being affected, while supply chain and remote attacks could lead to global-scale impact. Depending on the type and scale of a threat or exploit, hundreds to millions of people’s data could be compromised.

### Personnel and Physical Access

Unauthorized physical access or insider threats could allow for theft of assets or the use of other attack vectors on any phase of the ecosystem. Small independent laboratories do not often have resources to implement strong physical security. Large institutions are usually enabled to maintain strong physical security, but the relatively large number of personnel and devices that need to be secured creates a complex attack surface. Ultimately, the strongest cybersecurity can be easily circumvented by weak physical security.

Insider threats are a problem for information security because personnel possess deeper knowledge of an organization and its systems. Many countries rely on foreign nationals working in biotechnological fields that may be susceptible to foreign influence^19^, and citizens can also be susceptible^20^. Personnel could introduce many exploits on-site if coerced or threatened (Reed and Dunaway, 2019; Walsh and Streilein, 2020). Even when not acting in a purposefully malicious manner, personnel can unintentionally compromise the integrity and availability of genetic information through error (US Office of the Inspector General, 2004). Appropriate safeguards should be in place to ensure that privileged individuals are empowered to do their work correctly and efficiently, but all activities should be documented and monitored when working with sensitive genetic information.

### Biological Samples, Metadata, and Repositories

Sample collection, storage, and distribution processes have received little recognition as legitimate points for the compromise of genetic information. Biological samples as inputs into this ecosystem can be modified maliciously to contain encoded malware, although this has to date only been demonstrated in a system in which the sequencing software was artificially engineered to include a vulnerability that would be triggered by the encoded malware (Ney et al., 2017). Biological samples could also be degraded, modified, or destroyed to compromise the materials,’ and resulting data’s, integrity and availability. We found sample repository and storage equipment to often be connected to networks for monitoring purposes, making them vulnerable to adjacent network and remote attacks. Biorepositories and the collection and distribution of samples could be targeted to steal numerous biological samples, such as in known genetic testing scams^21^, and targeted exfiltration of small numbers of samples may be difficult to detect. The storage, transit, and destruction of sensitive biological material should be considered by stakeholders to be an important facet of overall genetic information security and cyberbiosecurity.

Though potentially unlikely, other organizations within the ecosystem could be targeted for the theft of samples and processed DNA libraries, as well. The wet laboratory preparation and DNA sequencing subphases last several weeks and produce unused waste and stored material. At the conclusion of sequencing runs, the consumables that contain DNA molecules are not always considered sensitive and can be found unwittingly maintained in many laboratories. Several cases have been documented of DNA being recovered and successfully sequenced while aged for years in non-controlled environments (Colotte et al., 2011). Limited attention is payed to the secure destruction of consumables or other potential sources of biological material as there is little concern for such targeted attacks.

### Laboratories and Equipment

DNA sequencing systems and laboratories were found to be multifaceted in their design and threat profile. DNA sequencing instruments have varying scalability of throughput, cost, and unique considerations for secure operation (Table 1). They have built-in computers and commonly have connected computers and servers for data storage, networking, and analytics. Sequencing system devices contain a number of different hardware components, firmware, software, and operating systems, including insecure legacy versions (National Academies of Sciences, Engineering, and Medicine, 2020). Wireless or wired local network and remote Internet connections are required for maintenance, data transmission, and analytics in most operations. Wireless capabilities and Bluetooth technology were commonly found within laboratories, presenting unnecessary access vectors and threats to these systems.

**TABLE 1:** Overview of popular genetic sequencing devices and systems.

**Vendor**	**Product**	**Time (h)**	**Output (Gb)**	**Operating system**	**Computing**	**Network connection**	**Cloud service (CSP)**
Illumina	iSeq	19	1	Windows 10 & Windows 7	Standalone &/or external device	Wired or wireless	BaseSpace (AWS)
	MiniSeq	24	8				
	MiSeq	24	15				
	NextSeq	30	300				
	HiSeq	84	1,500				
	NovaSeq	44	6,000				
Oxford Nanopore Technologies	SmidgION^M^	–	∼1	Android & iOS	External device	Wired or wireless	EPI2ME (AWS)
	Flongle^M^	16	2	Windows, Macintosh, Linux	External device	Wired	
	MinION Mk1B^M^	48	30				
	MinION Mk1C^M^	48	30	Linux (Ubuntu)	Standalone &/or external device		
	GridION Mk1	48	150				
	PromethION	72	8,600				
Pacific Biosciences	Sequel	20	50	Linux (Ubuntu & CentOS)	Standalone	Wired	SecureLink (AWS)
	Sequel II	30	4,000				
Applied Biosystems*	SeqStudio	2	∼0.45	Windows 10	Standalone & external device	Wired or wireless	Thermo Fisher Cloud (AWS)
	3500/3500xL	2	–	Windows Vista SP1			
	3730/3730xL	3	–	Windows 2000 Pro			
Ion Torrent*	GeneStudio S5	8	50	Linux (Ubuntu)	Standalone & external device	Wired	
	Genexus	48	20				

Maximum run time in hours, maximum output in gigabytes, operating system, computing capabilities, network connection type, and cloud platform provided per vendor and product. Time and output are maximum values based on one full sequencing run. Information gathered from vendors’ websites (https://www.illumina.com/systems/sequencing-platforms.html, https://nanoporetech.com/products/, https://www.pacb.com/products-and-services/, https://www.thermofisher.com/us/en/home/brands/applied-biosystems.html, https://www.thermofisher.com/us/en/home/brands/ion-torrent.html) and technical documentation (Supplementary Appendix 3). h, hours; Gb, gigabytes; CSP, cloud service provider; AWS, Amazon Web Services. *Thermo Fisher Scientific brands; ^*M*^Mobile sequencing instrument for in-field use.

Device vendors obtain various internal hardware components from several sources and integrate them into laboratory devices that contain vendor-specific intellectual property and software. Generic hardware components are often produced in various countries, which is cost effective but leads to insecurities and a lack of hardening for specific end-use purposes. Hardware vulnerabilities could be exploited on-site, or they can be implanted during manufacturing and supply-chain processes for widespread and unknown security issues (Anderson and Kuhn, 1997; Shwartz et al., 2017; Ender et al., 2020; Fayans et al., 2020). Such hardware issues are unpatchable and will remain with devices forever until newer devices can be manufactured to replace older versions. Unfortunately, adversaries can always shift their techniques to create novel vulnerabilities within new hardware in a continual vicious cycle.

Third-party manufacturers and device vendors implement firmware in these hardware components. Embedded device firmware has been shown to be more susceptible to cyber-attacks than other forms of software (Shwartz et al., 2017). In-field upgrades are difficult to implement, and like hardware, firmware and operating systems can be maliciously altered within the supply chain (Fayans et al., 2020). A firmware-level exploit would allow for the evasion of operating system and software-level security features. Firmware exploits can remain hidden for long periods, even after hardware replacements or wiping and restoring to default factory settings. For example, operating systems have specific disclosed Common Vulnerabilities and Exposures (CVEs)^22^. Additionally, researchers have confirmed the possibility of index hopping, or index misassignment, by sequencing device software, resulting in customers receiving confidential data from other customers (Ney et al., 2017) or downstream data processors inputting incorrect data into their analyses. Some software vulnerabilities can be partially mitigated by frequent updates. However, operating systems and firmware are typically updated every 6–12 months by a field agent accessing a sequencing device on site. Device operators are not allowed to modify the device in any way, yet they are responsible for security aspects of this equipment. With ubiquitous implementation throughout the ecosystem, software issues are especially concerning (National Academies of Sciences, Engineering, and Medicine, 2020).

DNA sequencing infrastructure is proliferating, and sequencing services are becoming more affordable. In 2020, technology developed by Beijing Genomics Institute has finally resulted in the $100 human genome (Drmanac, 2020) while US prices remain around $1,000 per sample. Stakeholders often take advantage of cheaper services by third-party sequencing providers that reside across national borders (Office of the US Trade Representative, 2018), indicating that genetic data could be aggregated globally by nation-states^23^ and other actors *during* the analysis phase.

### Storage and Compute Infrastructure

Raw signal sequencing data are stored on a sequencing system’s memory and are transmitted to one or more endpoints. Transmitting data securely across a local network requires internal information technology (IT) configurations. Vendor documentation usually mentions implementing a firewall to secure sequencing systems. Doing so correctly requires deep knowledge of secure networking and vigilance of network activity. Documentation also commonly mentions disabling and enabling certain network protocols and ports and further measures that can be difficult for most small- to medium-sized organizations, while also omitting other common controls and mitigations.

Laboratories and DNA sequencing systems are connected to third-party services, and laboratories have little control over the security posture of these connections. Independent cloud platforms and DNA sequencing vendors’ cloud platforms are implemented for bioinformatic processing, data storage, and device monitoring and maintenance capabilities (Table 1). Multi-factor authentication, role- and task-based access, and many other security measures are not common in these platforms. Misconfigurations to cloud services and remote communications are a primary vulnerability to genetic information, demonstrated by prior breaches. Laboratory information management systems (LIMS) are also frequently implemented within laboratories and connected to sequencing systems and laboratory networks (Roy et al., 2016), and DNA sequencing vendors provide their own LIMSs as part of their cloud offerings. Even when LIMS and cloud platforms meet all regulatory requirements for data security and privacy, they are handling data that is not truly anonymized and therefore remains identifiable and sensitive. Furthermore, specific CVEs have been disclosed for dnaTools’ dnaLIMS product^24^ that were actively exploited by a foreign nation-state^25^. Phishing attacks are another major threat, as email services add to the attack surface in many ways. Sequencing service providers often share links granting access to datasets via email. These email chains are a primary trail of transactions that could be exploited to exfiltrate data on clients, metadata of samples, or genetic data itself.

Some laboratories transmit raw data directly to an external hard drive per customer or regulatory requirements. Reducing network activity in this way can greatly minimize the threat surface of sensitive genetic information. Separating networks and devices from other networks, or air gapping, while using hard drives is possible, but even air-gapped systems have been shown to be vulnerable to compromise (Guri et al., 2019; Guri, 2020). Sequencing devices are still required to be connected to the Internet for maintenance and are often connected between offline operations. Hard drives can be physically secured and transported; however, these methods are time and resource intensive, and external drives could be compromised for the injection of modified software or malware.

### Bioinformatic Pipeline

To determine the success of a sequencing run, bioinformatics analyses are necessary, but this software has not been commonly scrutinized in security contexts or subjected to the same adversarial pressure as other more mature software (National Academies of Sciences, Engineering, and Medicine, 2020). Open-source software is widely used across genomics, acquired from several online code repositories, and heavily modified for individual purposes, but it is only secure when security researchers are incentivized to assess these products. In a specialized and niche industry like genomics and bioinformatics, this is typically not the case. Bioinformatic programs have been found to be vulnerable due to poor coding practices, insecure function usage, and buffer overflows (Ney et al., 2017), such as the Burrow Wheeler Aligner (BWA) example^26^ ^,27^. This program is hosted on cloud platforms and available for on-site use within laboratories. Researchers have also uncovered that algorithms can be forced to mis-classify by intentionally modifying data inputs, breaking the integrity of any resulting outputs (Finlayson et al., 2019). Nearly every imaginable algorithm, model type, and use case have been shown to be vulnerable to this kind of attack across many data types (Biggio and Roli, 2018), especially those relevant to raw signal and sequencing data formats. Similar attacks could be carried out in the processing of raw signal data internal to a sequencing system or on downstream bioinformatic analyses accepting raw sequencing data or processed data as an input.

### Dissemination Practices and Database Storage

Alarming amounts of human and other sensitive genetic data are publicly available^28^ ^,29^ ^,30^ ^,31^ ^,32^ (Vinatzer et al., 2019). Several funding and publication agencies require public dissemination, so researchers commonly contribute to open and semi-open databases (Shi and Wu, 2017). Healthcare providers either house their own internal databases or disseminate to third-party databases. Their clinical data is protected like any other healthcare information as required by regulations; however, this data can be sold and aggregated by external entities. DTC companies keep their own internal databases closely guarded and can charge steep prices for third-party access. Data sharing is prevalent when the price is right. Data originators often have access to their genetic data and test results for download in plaintext. These reports can then be uploaded to public databases, such as GEDmatch and DNA.Land, for further analyses, including finding distant genetic relatives with a shared ancestor (Erlich et al., 2018). A well-known use of such identification tactics was the infamous Golden State Killer case (Edge and Coop, 2019). Data sharing is dependent upon the data controller’s wants and needs, barring any legal or business requirements from other involved stakeholders.

Genetic database vulnerabilities have been well studied and disclosed (Gymrek et al., 2013; Erlich and Narayanan, 2014; Naveed et al., 2015; Edge et al., 2017; Ney et al., 2018; Vinatzer et al., 2019; Edge and Coop, 2020; Ney et al., 2020). For example, the contents of the entire GEDmatch database could be leaked by uploading artificial genomes (Ney et al., 2020). Such an attack would violate the confidentiality of more than a million users’ and their relatives’ genetic data because the information is not truly anonymized. Even social media posts can be filtered for keywords indicative of participation in genetic research studies to identify research participants in public databases (Liu et al., 2019). All told, tens of millions of research participants, consumers, and relatives may already be at risk.

## Discussion

Security is a spectrum; stakeholders must do everything they can to chase security as a best practice. Securing genetic information is a major challenge in this rapidly evolving ecosystem. Attention has primarily been placed on the post-analytical phase of genetic information systems for security and privacy, but adequate measures have yet to be universally adopted. The pre-analytical and analytical phases are also vulnerable points for data compromise that must be addressed. Adequate national regulations are needed for security and privacy enforcement, incentivization, and liability, but legal protection is dictated by regulators’ responses and timelines. However, data originators, controllers, and processors can take immediate action to protect their data.

Genetic information security is a shared responsibility between sequencing laboratories and device vendors, as well as all other involved stakeholders. To protect genetic information, laboratories, biorepositories, and other data processors need to create strong organizational policies and reinvestments toward their physical and cyber infrastructure. They also need to determine the sensitivity of their data and material and take necessary precautions to safeguard sensitive genetic information. Data controllers, especially healthcare providers and DTC companies, should reevaluate how their genetic data is generated and processed, with special consideration for the identifiability of human genetic data. Device vendors need to consider security when their products are being designed, implemented, and maintained throughout their lifecycles.

Many of these recommendations go against the current paradigms in genomics and related industries and will therefore take time, motivation, and incentivization before being actualized, with regulation being a critical factor. In order to secure and protect all stakeholders of genetic information systems, sequencing instrumentation, bioinformatics software, cloud platforms, data access models, and other system components need to be analyzed, and in-depth assessments of this threat surface will be required. Unique threat models and assessment frameworks are needed for specific and niche industry sectors, and genomics is a perfect example. Novel security and privacy countermeasures will need to be developed that protect the confidentiality of genetic information while ensuring its integrity for accurate diagnoses and applications and its availability for rapid public health responses. These security requirements will need to be balanced and dependent upon the context of use cases. These items will require collaborative engagement between stakeholders to reevaluate and implement improved security controls into genetic information systems (Berger and Schneck, 2019; Schabacker et al., 2019; Moritz et al., 2020). The development and implementation of genetic information security will foster a healthy and sustainable bioeconomy without damaging privacy or security.

There can be security without privacy, but privacy requires security. These two can be at odds with one another in certain contexts. For example, personal security aligns with personal privacy, whereas public security can require encroachment on personal privacy. A similar story is unfolding within genomics. Genetic data must be shared for public good, but this can jeopardize personal privacy. However, genetic data necessitates the strongest protections possible for public security and personal security. Appropriate genetic information security will simultaneously protect everyone’s safety, health, *and* privacy.

## Data Availability Statement

The datasets presented in this article are not readily available because the interviewed and assessed stakeholders involved in this work have requested anonymity and confidentiality due to their service agreements with vendors and the sensitivity of the information they supplied. Ethical vulnerability disclosures are ongoing with vendors and the US Cybersecurity and Infrastructure Security Agency that will be published in future manuscripts when appropriate to do so. Therefore, limited data is available beyond the findings presented within the manuscript and accompanying Supplementary Material. Requests to access the datasets should be directed to GS, g@geneinfosec.com.

## Author Contributions

GS: inception and drafting of manuscript. GS, SS, and DK: literature review and analysis. GS, SS, DK, and AH: stakeholder engagement, interviews, and critical review of draft. GS and AH: security assessments. All authors contributed to the article and approved the submitted version.

## Conflict of Interest

GS, SS, and DK were founders and owners of GeneInfoSec Inc. and are developing technology and services to protect genetic and other biological information systems. GeneInfoSec Inc. has not received US Federal research funding. AH when writing this manuscript and submitting it to the bioRxiv preprint server, declared that the research was conducted in the absence of any commercial or financial relationships that could be construed as a potential conflict of interest. AH now declares a potential future interest as a consultant in the area of laboratory information security.

## Footnotes

1
https://www.bloomberg.com/news/articles/2020-08-05/blackstone-said-to-reach-4-7-billion-deal-to-buy-ancestry-com

2
https://www.gsk.com/en-gb/media/press-releases/gsk-and-23andme-sign-agreement-to-leverage-genetic-insights-for-the-development-of-novel-medicines/

3
https://www.ancestry.com/corporate/newsroom/press-releases/ancestrydna-and-calico-to-research-the-genetics-of-human-lifespan

4
https://www.bloomberg.com/news/articles/2019-11-06/breach-at-dna-test-firm-veritas-exposed-customer-information

5
https://www.bostonherald.com/2019/08/22/mgh-data-breach-exposes-10000-patients/

6
https://www.latimes.com/business/la-fi-vitagene-dna-privacy-exposed-20190709-story.html

7
https://www.komando.com/security-privacy/ancestry-com-suffers-big-data-leak-300000-user-credentials-exposed/435921/

8
https://www.wizcase.com/blog/mackiev-leak-research/

9
https://blog.myheritage.com/2020/07/security-alert-malicious-phishing-attempt-detected-possibly-connected-to-gedmatch-breach/

10
https://www.ambrygen.com/legal/substitute-notice

11
https://media.dojmt.gov/wp-content/uploads/Data-Breach-NotificationDetails11.pdf

12
https://media.dojmt.gov/wp-content/uploads/Consumer-Notice-73.pdf

13
https://privacyrights.org/data-breaches/myriad-genetic-laboratories-inc

14
https://blog.myheritage.com/2018/06/myheritage-statement-about-a-cybersecurity-incident/

15
https://media.dojmt.gov/wp-content/uploads/Shire-Human-Genetic-Therapies-Inc.pdf

16
https://www.classaction.org/news/ambry-genetics-corp-hit-with-class-action-over-jan-2020-data-breach-affecting-230000

17
https://www.telegraph.co.uk/news/2018/12/05/nhs-storing-patients-genetic-data-high-security-army-base-due/

18
https://www.telegraph.co.uk/technology/2020/03/09/dna-testing-firms-risk-state-sponsored-hacks-says-23andme-security/

19
https://www.fbi.gov/investigate/counterintelligence/foreign-influence

20
https://www.sciencemag.org/news/2020/06/fifty-four-scientists-have-lost-their-jobs-result-nih-probe-foreign-ties

21
https://oig.hhs.gov/fraud/consumer-alerts/alerts/geneticscam.asp

22
https://cve.mitre.org/

23
https://www.fbi.gov/news/pressrel/press-releases/fbi-and-cisa-warn-against-chinese-targeting-of-covid-19-research-organizations

24
https://www.shorebreaksecurity.com/blog/product-security-advisory-psa0002-dnalims/

25
https://www.zdnet.com/article/mysterious-iranian-group-is-hacking-into-dna-sequencers/

26
https://share-ng.sandia.gov/news/resources/news_releases/genomic_cybersecurity/

27
https://nvd.nist.gov/vuln/detail/CVE-2019-10269

28
https://my.pgp-hms.org/public_genetic_data

29
https://gnomad.broadinstitute.org/downloads

30
https://platform.stjude.cloud/data/diseases

31
https://www.ensembl.org/Homo_sapiens/Info/Index

32
https://www.completegenomics.com/public-data/
