# Supplementary material for: Genetic Information Insecurity as State of the Art
Source: Front Bioeng Biotechnol. 2020 Dec 8;8:591980. doi: 10.3389/fbioe.2020.591980 (PMC7768984; doi:10.3389/fbioe.2020.591980)
Supplement: Supplementary file 1 [file Data_Sheet_1.DOCX]

*Supplementary Material*

# Appendix 1. Overview of Genetics/Genomics Stakeholders

Genomics stakeholders are categorized based upon their influence, contributions, and handling of biological samples and resulting genetic data (Supplementary Table 1). Asymmetries exist between stakeholders in these regards^^[[1]](#footnote-1)^^. *Data originators* are humans that voluntarily or involuntarily are the source of biological samples or are investigators collecting samples from non-human specimens. Examples of data originators include consumers, healthcare patients, military personnel, research subjects, migrants, criminals, and their relatives. *Data controllers* are entities that are legally liable for and dictate the use of biological samples and resulting data. In human-derived contexts, data controllers are typically healthcare providers, researchers, law enforcement agencies, or DTC companies. *Data processors* are entities that collect, store, generate, analyze, disseminate, and/or apply biological samples or genetic data. Data processors may also be data originators and data controllers. Examples include biorepositories, DNA sequencing laboratories, researchers, cloud and other service providers, and supply chain entities responsible for devices, software and materials. *Regulators* oversee this ecosystem and the application and use of biotechnology, biological samples, genetic data, and market/industry trends at the transnational, national, local, and organizational levels.

**Supplementary Table 1.** Categories and groups of genetics/genomics ecosystem stakeholders with examples of organizations and contexts.

| Stakeholder | Group | Examples / context |
| --- | --- | --- |
| Data originators | Consumers, patients, subjects | Humans voluntarily providing biological samples |
|  | Researchers | Investigators collecting non-human samples |
|  | Suspected criminals, migrants, relatives | Humans involuntarily providing biological samples |
| Data controllers | Data originators | Rare instances of individual data control, e.g., Genetic Alliance |
|  | DTC companies | 23andme, Ancestry.com, FamilyTreeDNA, MyHeritage |
|  | Healthcare providers | Kaiser, UnitedHealth, NorthShore, Veterans Affairs (VA) |
|  | Research organizations | Academic, military, government and industry groups |
| Data processors | Biorepositories / biobanks | All of Us (NIH), UK Biobank, Beryllium BioBank |
|  | Cloud service providers | AWS, Google Cloud, Box, LifeOmic, BioBright, BlueBee |
|  | Data controllers | DTC, healthcare & research groups processing data |
|  | Device manufacturers/vendors | Microsoft, Apple, Dell, Linux-based groups, Agilent, Qiagen |
|  | Diagnostics providers | Quest Diagnostics, Ambry Genetics, academic laboratories |
|  | Email service providers | Gmail, Microsoft Outlook, ProtonMail |
|  | IT organizations | Internal and third-party IT services, Internet providers |
|  | Open and semi-open databases | GEDmatch, All of Us (NIH), Genomics England (NHS) |
|  | Sequencing laboratories | Third-party and internal sequencing service providers |
|  | Sequencing system vendors | Illumina, Oxford Nanopore, Thermo Fisher, Pacific Biosciences |
|  | Synthetic DNA providers | Ginkgo, IDT, Agilent, Twist Biosciences, Thermo Fisher |
| Regulators | Consumers | Dictate aspects of ecosystem through behavior |
|  | Federal organizations | US Centers for Medicare & Medicaid Services, FDA |
|  | International and national governments | United States, European Union, United Kingdom |
|  | Intranational governments | Providences, states, counties, territories, tribes |
|  | Law enforcement | FBI, DHS, DOJ, military, police |
|  | Organizational policy creators | Management and executive personnel |

Acronyms: DTC, direct-to-consumer; NIH, US National Institutes of Health; UK, United Kingdom; IT, information technology; NHS, UK National Health Services; IDT, Integrated DNA Technologies; US, United States; FDA, US Food and Drug Administration; FBI, US Federal Bureau of Investigation; DHS, US Department of Homeland Security; DOJ, US Department of Justice.

# Appendix 2. Overview of the Genetic Information System Phases

## Appendix 2.1 Pre-Analytical Phase

Biological samples and metadata from the samples must first be collected once a data originator or controller determines to proceed with genetic analysis. Biological samples can be sourced from any biological entity relying on nucleic acids for reproduction, replication, and other processes, including non-living microbes (e.g., viruses, prions), microorganisms (e.g., bacteria, fungi), and organisms (e.g., plants, animals). Samples are typically de-identified of metadata and given a numeric identifier, but this is largely determined by the interests of data controllers and the regulations that may pertain to various sample types. Metadata includes demographic details, inclusion and exclusion criteria, pedigree structure, health conditions critical for secondary analysis, and other identifying information^^[[2]](#footnote-2)^^. It can also be in the form of quality metrics obtained during the analysis phase. Samples are then stored in controlled environments at decreased temperature, moisture, light, and oxygen to avoid degradation. Sample repositories can be internal or third-party infrastructure housing small to extremely large quantities of material for short- and long-term storage. Following storage, samples are distributed to an internal or third-party laboratory for DNA sequencing preparations.

## Appendix 2.2 Analytical Phase

The analytical phase is divided into three subphases: wet laboratory preparation, DNA sequencing, and bioinformatic pipeline.

### Appendix 2.2.1 Wet Laboratory Preparation

The wet laboratory preparation phase chemically prepares biological samples for sequencing with sequencing-platform-dependent methods. This phase can be performed manually with time- and labor-intensive methods, or it can be highly automated to reduce costs, run-time, and error. Common initial preparation steps involve removing contaminants and unwanted material from biological samples and extracting and purifying samples’ nucleic acids. If RNA is to be sequenced, it is usually converted into complementary DNA. Once DNA has been isolated, a library for sequencing is created via size-selection, sequencing adapter ligation, and other chemical processes. Adapters are synthetic DNA molecules attached to DNA fragments for sequencing and contain sample indexes, or identifiers. Indexes allow for multiplexing sequencing runs with many samples at once to increase throughput, decrease costs, and to identify DNA fragments to their sample source.

### Appendix 2.2.2 DNA Sequencing

To begin sequencing, prepared libraries are loaded into a DNA sequencing instrument with the required materials and reagents. Laboratory personnel must login to the instrument and any connected services, such as cloud services or information management systems, and configure a run to initiate sequencing. A single sequencing run can generate gigabytes to terabytes of raw sequencing data and last anywhere from a few hours to multiple days, requiring the devices to commonly be left unmonitored during operation. Raw data can be stored on the instrument's local memory and are transmitted to one or more of the following endpoints during or following a sequencing run: (i) local servers, computers, or other devices within the laboratory; (ii) cloud services of the vendor or other service providers; and (iii) external hard drives directly tethered to the sequencer. Data paths largely depend on the sequencing platform, the laboratory's capabilities and infrastructure, and the sensitivity of data being processed. Certain regulations require external hard drive use and offline data storage, analysis, and transmission.

### Appendix 2.2.1 Bioinformatic Pipeline

Bioinformatic pipelines convert raw data through a series of software tools into usable forms. Raw signal data include images, chemical signal, electrical current signal, and other forms of signal data dependent upon the sequencing platform. Primary analyses convert raw signal data into sequence data with accompanying quality metrics through a process known as basecalling. Many sequencing instruments can perform these functions. The length of each DNA molecule sequenced is orders of magnitude smaller than genes or genomes of interest, so basecalled sequence data must then be aligned to determine each read’s position within a genome or genomic region. This aligned sequence data is then compared to reference genomes sourced from databases through a procedure known as variance detection to determine differences between a sample’s data and the accepted normal genomic sequence. Only the unique genetic variants of a sample are retained in variance call format (VCFs) files, a common final processed data form. VCF files are vastly smaller than the gigabytes to terabytes of raw data initially produced, making them an efficient format for long-term storage, dissemination, and analysis purposes. However, this file format exists as a security threat for sensitive genetic data because these files are personally identifiable and contain sensitive health information.

### Appendix 2.3 Post-Analytical Phase

Following data analyses, processed data are integrated with metadata and ultimately interpreted for the data controller’s purpose. Metadata and genetic data are often housed together, and exploiting this combined information could lead to numerous risks and threats to the data originators, their relatives, and the liable entities involved along the data path. Secondary analyses can be performed on datasets by data controllers and third-party data processors to answer any number of relevant research questions, such as in diagnostics or ancestry analysis. Genetic research is only powerful when large datasets are created containing numerous data points from thousands to millions of samples. Therefore, genetic data is widely distributed and accessible via remote means across numerous databases and stakeholders.

# Appendix 3. Genetic Sequencing System Vendor Documentation

Applied Biosystems, Life Technologies Corp. (Hitachi), Thermo Fisher Scientific, Inc. Applied Biosystems 3500/3500xL Genetic Analyzer User Guide. (2010). *Thermo Fisher Scientific publication #4401661, Rev. C*. Retrieved from<http://tools.thermofisher.com/content/sfs/manuals/4401661.pdf>.

Applied Biosystems, Life Technologies Corp. (Hitachi), Thermo Fisher Scientific, Inc. Applied Biosystems 3730/37300xL DNA Analyzers User Guide. (2010). *Thermo Fisher Scientific publication #444331468, Rev. E*. Retrieved from<https://assets.thermofisher.com/TFS-Assets/LSG/manuals/cms_041259.pdf>.

Applied Biosystems, Thermo Fisher Scientific, Inc. Applied Biosystems SeqStudio Genetic Analyzer Specification Sheet. (2020). *Thermo Fisher Scientific publication #COL23988 0320*. Retrieved from<https://assets.thermofisher.com/TFS-Assets/GSD/Specification-Sheets/SeqStudio-Specification-Sheet.pdf.pdf>.

Illumina, Inc. HiSeq X System Lab Setup and Prep Guide. (January 2017). *Illumina document #15050093 v05*. Retrieved from<https://support.illumina.com/content/dam/illumina-support/documents/documentation/system_documentation/hiseqx/hiseq-x-lab-setup-and-site-prep-guide-15050093-05.pdf>.

Illumina, Inc. Illumina Proactive | Data Security Sheet. (2019). *Illumina document #970-2019-019-A*. Retrieved from<https://www.illumina.com/content/dam/illumina-marketing/documents/informatics/illumina-proactive-data-security-sheet-970-2019-019.pdf>.

Illumina, Inc. iScan System Site Prep Guide. (January 2019). *Illumina document #1000000000661 v01*. Retrieved from<https://support.illumina.com/content/dam/illumina-support/documents/documentation/system_documentation/iscan/iscan-system-site-prep-guide-1000000000661-01.pdf>.

Illumina, Inc. iSeq 100 Sequencing System Site Prep Guide. (April 2020). *Illumina document #1000000035337 v07*. Retrieved from<https://support.illumina.com/content/dam/illumina-support/documents/documentation/system_documentation/iseq100/iseq-100-site-prep-guide-1000000035337-07.pdf>.

Illumina, Inc. MiniSeq System Guide. (February 2020). *Illumina document # 1000000002695 v03, Material #20014309*. Retrieved from<https://support.illumina.com/content/dam/illumina-support/documents/documentation/system_documentation/miniseq/miniseq-system-guide-1000000002695-03.pdf>.

Illumina, Inc. MiSeq System Guide. (August 2019). *Illumina document #15027617 v05, Material #20000262*. Retrieved from<https://support.illumina.com/content/dam/illumina-support/documents/documentation/system_documentation/miseq/miseq-system-guide-for-local-run-manager-15027617-05.pdf>.

Illumina, Inc. NextSeq 550Dx Instrument Site Prep Guide. (March 2019). *Illumina document #1000000009869 v03*. Retrieved from<https://support.illumina.com/content/dam/illumina-support/documents/documentation/system_documentation/nextseq-550dx/nextseq-550dx-instrument-site-prep-guide-1000000009869-03.pdf>.

Illumina, Inc. NovaSeq 6000 Sequencing System Site Prep Guide. (January 2019). *Illumina document #1000000019360 v06*. Retrieved from<https://support.illumina.com/content/dam/illumina-support/documents/documentation/system_documentation/novaseq/novaseq-site-prep-guide-1000000019360-06.pdf>.

Ion Torrent, Thermo Fisher Scientific, Inc.. Ion GeneStudio S5 Series Specification Sheet. (2018). *Thermo Fisher Scientific publication #COL22253 0118*. Retrieved from<https://assets.thermofisher.com/TFS-Assets/CSD/Specification-Sheets/PG1720-PJT2769-COL22253-P-on-GeneStudio-S5-Spec-Sheet-Global-FLR.pdf>.

Ion Torrent, Thermo Fisher Scientific, Inc.. Ion GeneStudio S5 Series Systems Brochure. (2018). *Thermo Fisher Scientific publication #COL06368 1018*. Retrieved from<https://assets.thermofisher.com/TFS-Assets/CSD/brochures/ion-genestudio-s5-ngs-system-brochure.pdf>.

Ion Torrent, Thermo Fisher Scientific, Inc.. Ion Torrent Genexus Integrated Sequencer Performance Summary Sheet. (2019). *Thermo Fisher Scientific publication #MAN0017918*. Retrieved from<https://assets.thermofisher.com/TFS-Assets/LSG/manuals/MAN0017918_GenexusIntegratedSequencer_SPG.pdf>.

Oxford Nanopore Technologies. GridION Mk1 Site Installation and Device Operation Requirements, version 1. (July 2019). Retrieved from<https://nanoporetech.com/sites/default/files/s3/products/GridION%20Mk1%20IT%20Requirements_v1.pdf>.

Oxford Nanopore Technologies. MinION IT requirements, version 3. (February 2020). Retrieved from<https://community.nanoporetech.com/requirements_documents/minion-it-reqs.pdf>.

Oxford Nanopore Technologies. PromethION P24/P48 Site Installation and Device Operation Requirements, version 1. (July 2019). Retrieved from<https://nanoporetech.com/sites/default/files/s3/products/PromethION%20P24-P48%20IT%20Requirements_v1.pdf>.

Pacific Biosciences of California, Inc. Operations Guide – Sequel System: The SMRT Sequencer. (October 2018). *PacBio publication #101-055-100-06*. Retrieved from<https://www.pacb.com/wp-content/uploads/Operations-Guide-The-SMRT-Sequencer-Sequel-System.pdf>.

Pacific Biosciences of California, Inc. Operations Guide – Sequel II System: The SMRT Sequencer. (April 2019). *PacBio publication #101-774-700-01*. Retrieved from<https://www.pacb.com/wp-content/uploads/Operations-Guide-Sequel-II-System.pdf>.

Thermo Fisher Scientific, Inc. Security Operations Guide: Connect Platform | IoT Connectivity, version 4.2. (2019). Retrieved from<https://assets.thermofisher.com/TFS-Assets/CORP/Reference-Materials/Connect_whitepaper_Nov2019.pdf>.

1. Berger, K. M., & Schneck, P. A. (2019). National and transnational security implications of asymmetric access to and use of biological data. *Frontiers in bioengineering and biotechnology, 7*, 21. [↑](#footnote-ref-1)
2. Erlich, Y., & Narayanan, A. (2014). Routes for breaching and protecting genetic privacy. *Nature Reviews Genetics, 15*(6), 409-421. [↑](#footnote-ref-2)
